# Evaluation of common genetic variants in 82 candidate genes as risk factors for neural tube defects

**DOI:** 10.1186/1471-2350-13-62

**Published:** 2012-08-02

**Authors:** Faith Pangilinan, Anne M Molloy, James L Mills, James F Troendle, Anne Parle-McDermott, Caroline Signore, Valerie B O’Leary, Peter Chines, Jessica M Seay, Kerry Geiler-Samerotte, Adam Mitchell, Julia E VanderMeer, Kristine M Krebs, Angelica Sanchez, Joshua Cornman-Homonoff, Nicole Stone, Mary Conley, Peadar N Kirke, Barry Shane, John M Scott, Lawrence C Brody

**Affiliations:** 1 Molecular Pathogenesis Section, Genome Technology Branch, National Human Genome Research Institute, National Institutes of Health, Bethesda, MD, USA; 2 Department of Clinical Medicine, Trinity College Dublin, Dublin, Ireland; 3 Division of Epidemiology, Statistics and Prevention Research, Eunice Kennedy Shriver National Institute of Child Health and Human Development, National Institutes of Health, Department of Health and Human Services, Bethesda, MD, USA; 4 Department of Health and Human Services, Office of Biostatistics Research, National Heart Lung and Blood Institute, National Institutes of Health, Bethesda, MD, USA; 5 School of Biotechnology, Dublin City University, Dublin, Ireland; 6 Department of Health and Human Services, Pregnancy and Perinatology Branch, National Institute of Child Health and Human Development, National Institutes of Health, Bethesda, MD, USA; 7 International Centre for Neurotherapeutics, Dublin City University, Dublin, Ireland; 8 Department of Health and Human Services, Molecular Genetics Section, Genome Technology Branch, National Human Genome Research Institute, National Institutes of Health, Bethesda, MD, USA; 9 Child Health Epidemiology Unit, Health Research Board, Dublin, Ireland; 10 Department of Nutritional Sciences and Toxicology, University of California, Berkeley, CA, 94720-3104, USA; 11 School of Biochemistry and Immunology, Trinity College Dublin, Dublin, Ireland; 12 Molecular Pathogenesis Section, Genome Technology Branch, National Human Genome Research Institute, Building 50, Room 5306, 50 South Drive, MSC 8004, Bethesda, MD, 20892-8004, USA

**Keywords:** Neural tube defects, Spina bifida, Folic acid, One-carbon metabolism, Candidate gene

## Abstract

**Background:**

Neural tube defects (NTDs) are common birth defects (~1 in 1000 pregnancies in the US and Europe) that have complex origins, including environmental and genetic factors. A low level of maternal folate is one well-established risk factor, with maternal periconceptional folic acid supplementation reducing the occurrence of NTD pregnancies by 50-70%. Gene variants in the folate metabolic pathway (e.g., *MTHFR* rs1801133 (677 C > T) and *MTHFD1* rs2236225 (R653Q)) have been found to increase NTD risk. We hypothesized that variants in additional folate/B12 pathway genes contribute to NTD risk.

**Methods:**

A tagSNP approach was used to screen common variation in 82 candidate genes selected from the folate/B12 pathway and NTD mouse models. We initially genotyped polymorphisms in 320 Irish triads (NTD cases and their parents), including 301 cases and 341 Irish controls to perform case–control and family based association tests. Significantly associated polymorphisms were genotyped in a secondary set of 250 families that included 229 cases and 658 controls. The combined results for 1441 SNPs were used in a joint analysis to test for case and maternal effects.

**Results:**

Nearly 70 SNPs in 30 genes were found to be associated with NTDs at the p < 0.01 level. The ten strongest association signals (p-value range: 0.0003–0.0023) were found in nine genes (*MFTC, CDKN2A, ADA, PEMT, CUBN, GART, DNMT3A, MTHFD1* and *T (Brachyury)*) and included the known NTD risk factor *MTHFD1* R653Q (rs2236225). The single strongest signal was observed in a new candidate, *MFTC* rs17803441 (OR = 1.61 [1.23-2.08], p = 0.0003 for the minor allele). Though nominally significant, these associations did not remain significant after correction for multiple hypothesis testing.

**Conclusions:**

To our knowledge, with respect to sample size and scope of evaluation of candidate polymorphisms, this is the largest NTD genetic association study reported to date. The scale of the study and the stringency of correction are likely to have contributed to real associations failing to survive correction. We have produced a ranked list of variants with the strongest association signals. Variants in the highest rank of associations are likely to include true associations and should be high priority candidates for further study of NTD risk.

## Background

Neural tube defects (NTDs) are one of the most common birth defects, with a historical prevalence of ~1 in 1000 in the US [1,2]. The NTD rate is now closer to ~5 in 10,000 in areas with folic acid fortification, such as the US [3] and many European countries [4]. Between 21 and 28 days after conception, the neural plate folds and closes to form the neural tube; this structure later develops into the brain and spinal cord. Failure of the neural tube to close most commonly leads to spina bifida or anencephaly, although encephalocele, craniorachischisis and iniencephaly can also occur [5].

It is known that both environmental and genetic factors contribute to the development of NTDs. The most established environmental factor is dietary folate; significantly lower levels of folate are observed in mothers with an NTD pregnancy [6], and periconceptional folate supplementation can reduce the risk of an NTD pregnancy by up to 75% [7][8][9]. There is also growing evidence of the importance of cobalamin (vitamin B12) in the etiology of NTDs. Like folate, lower vitamin B12 levels have been reported in mothers with an NTD pregnancy [6,10-17].

Genetic factors also contribute to NTDs. Compared to the general population, there is a 10–20 fold higher recurrence risk to siblings in families with an NTD child [18-20]. This, combined with the recognition of the importance of maternal folate, has led many groups to evaluate genetic polymorphisms related to the folate metabolic pathway as risk factors for NTDs. The best studied genetic risk factor is a single nucleotide polymorphism (SNP) in 5, 10-methylene-tetrahydrofolate reductase (*MTHFR*). The 677 C > T polymorphism results in the substitution of a valine for an alanine at codon 222 (A222V), leading to a thermolabile isoform of the protein [21]. A significantly higher frequency of the *MTHFR* 677 TT genotype has been observed in NTD cases in many populations (reviewed in [22]). Genetic variants associated with NTDs have been reported in other genes encoding folate- and vitamin B12-related proteins, such as methylenetetrahydrofolate dehydrogenase (NADP + dependent) 1, methenyltetrahydrofolate cyclohydrolase, formyltetrahydrofolate synthetase (*MTHFD1*) [23-26], methylenetetrahydrofolate dehydrogenase (NADP + dependent) 1-like (*MTHFD1L*) [27], dihydrofolate reductase (*DHFR*) [28,29], methionine synthase reductase (*MTRR*) [16,26,30-33], and the transcobalamin II receptor (*TCblR*) [34].

As genotyping technology has advanced, the scale of studies attempting to identify genetic NTD risk factors has grown from single SNP analyses to simultaneous evaluation of dozens of variants. Several studies have evaluated specific candidate polymorphisms with evidence of functional changes and/or disease risk (87 variants in 45 genes, [35]; 48 SNPs in 11 genes [36]; 64 SNPs in 34 genes [37]). In contrast, other studies have examined all common variation in candidate genes via tagging SNPs (tagSNPs) in specific genes of interest (118 tagSNPs in 14 folate-related genes [26]; 37 tagSNPs in 6 transcriptional activator genes [38]). In the current study, we also took the tagSNP approach to evaluate 1441 SNPs in 82 candidate genes for NTD risk.

## Results

**Figure 1:**
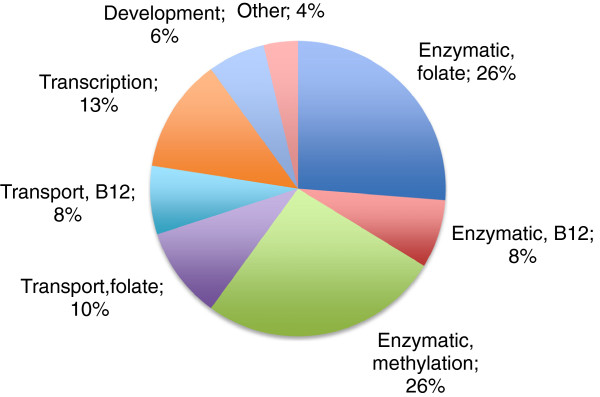
**Classification Distribution of 82 Selected Candidate Genes.** Categories of selected candidate genes (legend) and their distribution (pie chart) are shown. Over half of the genes selected for study are known to encode a protein with enzymatic function related to the folate/vitamin B12 pathway.

Common genetic variation in 82 candidate genes (Figure 1) was tested for association with NTDs. Results were generated in two stages. In the first stage, four broad tests of association were performed on all SNPs using a subset of samples. In the second stage, SNPs of interest identified in the initial analysis were then typed in the complete cohort to maximize the power to detect an effect, and a wider range of genetic models were applied to the combined dataset to evaluate the potential contribution of all SNPs to case or maternal risk of NTDs.

### Initial analyses

The primary sample set (320 NTD case families and 341 controls) was genotyped for 1517 tagging SNPs intended to capture common genetic variation in 82 candidate genes related to folate/vitamin B12 metabolism, transport of folate or vitamin B12, or transcriptional or developmental processes implicated in NTD mouse models (Table 1). Genotype data was successfully obtained for 1320 SNPs. Four tests of association were performed; two tests were to detect NTD case risk and two tests were to detect maternal risk for an NTD pregnancy. There were 203 SNPs in 54 genes that were significant (p < 0.05) by at least one test of association. A gene-based approach was used to select SNPs from fifteen genes to be genotyped in the secondary sample set. Five genes (mitochondrial folate transporter/carrier (*MFTC*), megalin (*LRP2*, low density lipoprotein receptor-related protein 2), DNA (cytosine-5-)-methyltransferase 3 beta (*DNMT3B*), phosphatidylethanolamine N-methyltransferase (*PEMT*), and euchromatic histone-lysine N-methyltransferase (*EHMT1*)) contained at least one SNP that was positive for both tests of a case effect or both tests of a maternal effect. An additional four genes (5-methyltetrahydrofolate-homocysteine methyltransferase 1 (*MTR*), cubilin (*CUBN*, intrinsic factor-cobalamin receptor), *T* (Brachyury homolog (mouse)), and AT rich interactive domain 1A (*ARID1A*)) contained more than five SNPs significant for any of the four tests of association. The remaining six genes (adenosine deaminase (*ADA*), ferritin, heavy polypeptide 1 (*FTH1*), cystathionase (*CTH*), peptidyl arginine deiminase, type IV (*PADI4*), low density lipoprotein receptor-related protein 6 (*LRP6*), and serine hydroxymethyltransferase 1 (*SHMT1*)) were selected based on a combination of factors, including the number of positive SNPs, their level of significance, and biological plausibility. Any SNP in these genes significant by any of the four tests of association (Table 2) was selected for genotyping in the secondary sample set (258 additional case families and 658 additional controls).

**Table 1:** Candidate Genes Selected for Evaluation of Common Genetic Variation as NTD Risk Factors

**Gene**	**Description**	**No. SNPs**	Category^*^
*ADA*	Adenosine deaminase	20	1 C
*AHCY*	S-Adenosylhomocysteine hydrolase	4	1 C
*ALDH1A2*	Aldehyde dehydrogenase 1 family, member A2	24	1A
*ALDH1L1*	Aldehyde dehydrogenase 1 family, member L1	35	1A
*AMN*	Amnionless homolog (mouse)	5	4
*ARID1A*	AT rich interactive domain 1A (SWI- like)	20	3
*ATIC*	5-aminoimidazole-4-carboxamide ribonucleotide formyltransferase/IMP cyclohydrolase	9	1A
*BAT8*	HLA-B associated transcript 8	8	5
*BHMT*	Betaine-homocysteine methyltransferase1	12	1 C
*BHMT2*	Betaine-homocysteine methyltransferase2	2	1 C
*CCL2*	Chemokine (C-C motif) ligand 2 (Monocyte chemoattractant protein 1)	6	5
*CDKN2A*	Cyclin-dep. kinase inhibitor 2A (melanoma, p16, inhibitor CDK4, P14arf)	10	3
*CITED2*	Cbp/p300-interacting transactivator, w. Glu/Asp-rich C-teminal domain, 2	6	3
*COMT*	Catechol-O-methyltransferase	16	1 C
*CREBBP*	CREB binding protein (Rubinstein-Taybi syndrome)	22	3
*CTH*	Cystathionase (cystathionine gamma-lyase)	16	1 C
*CUBN*	Cubilin (intrinsic factor-cobalamin receptor)	141	2B
*CYB5R2*	Cytochrome b5 reductase2	12	1A
*DHFR*	Dihydrofolate reductase	10	1A
*DMGDH*	Dimethylglycine dehydrogenase	24	1A
*DNMT1*	DNA cytosine 5 methyltransferase1	7	1 C
*DNMT3A*	DNA cytosine 5 methyltransferase3a	26	1 C
*DNMT3B*	DNA cytosine 5 methyltransferase3b	19	1 C
*EHMT1*	euchromatic histone methyltransferase 1	20	1 C
*ENOSF1*	Enolase superfamily member 1	22	1A
*EP300*	E1A binding protein p300	10	3
*EPHA3*	EPH (ephrin) receptor A3	45	4
*FOLH1*	Folylpolyglutamate hydrolase (gamma-glutamyl hydrolase, Glutamate carboxypeptidase II)	9	2A
*FOLR1*	Folate receptor alpha	7	2A
*FOLR2*	Folate receptor 2 (fetal)	5	2A
*FOLR3*	Folate binding protein gamma and g	8	2A
*FPGS*	Folylpolyglutamate synthase	4	2A
*FTCD*	Formiminoglutamate formiminotransferase-cyclodeaminase	17	1A
*FTH1*	Ferritin heavy chain	11	1A
*GART*	Phosphoribosylglycinamide formyltransferase/synthetase, phosphoribosylaminoimidazole synthetase	10	1A
*GGH*	Lysosomal gamma-glutamylhydrolase	11	1A
*GIF*	Intrinsic factor (gastric IF)	4	2A
*GNMT*	Glycine N-methyltransferase	5	1 C
*GRIK5*	Glutamate receptor, ionotropic, kainate 5	4	4
*HIF1A*	Hypoxia-inducible factor 1, alpha subunit (basic helix-loop-helix trx factor)	11	3
*HRMT1L2*	PRMT1 (Protein arginine methyltransferase 1) HMT1 hnRNA Mtase-like2	8	1 C
*HRMT1L3*	PRMT3, HMT1 hnRNA Mtase-like3	30	1 C
*ICMT*	Isoprenylcysteine carboxyl methyltranferase	7	1 C
*IRF6*	Interferon regulatory factor 6	11	3
*LRP2*	Megalin, low density lipoprotein-related protein 2	75	2B
*LRP6*	Low density lipoprotein receptor-related protein 6	24	2B
*MAT1A*	Methionine adenosyltransferase 1	19	1 C
*MAT2A*	Methionine adenosyltransferase II alpha	4	1 C
*MAT2B*	Methionine adenosyltransferase II beta	9	1 C
*MFTC*	Mitochondrial folate transporter/carrier	11	2A
*MMAA*	Methylmalonic aciduria (cobalamin deficiency) type A	14	1B
*MMAB*	AdoB12 adenosyltransferase, methylmalonic aciduria type B	7	1B
*MMACHC*	Methylmalonic aciduria cblC type with homocystinuria (CblC compl group)	8	1B
*MTHFD1*	Methylenetetrahydrofolate dehydrogenase (NADP + dependent) 1, methenyltetrahydrofolate cyclohydrolase, formyltetrahydrofolate synthetase (cytosolic)	20	1A
*MTHFD1L*	Methylenetetrahydrofolate dehydrogenase (NADP + dependent) 1-like (Formyltetrahydrofolate synthetase domain containing 1) (mitochondrial)	115	1A
*MTHFD2*	Methylenetetrahydrofolate dehydrogenase (NADP + dependent) 2, methenyltetrahydrofolate cyclohydrolase (mitochondrial)	6	1A
*MTHFR*	Methylenetetrahydrofolate reductase	19	1A
*MTHFS*	5,10-Methenyltetrahydrofolate synthetase	18	1A
*MTR*	Methionine synthase	20	1B
*MTRR*	Methionine synthase reductase	23	1B
*NDOR1*	NADPH dependent diflavin oxidoreductase 1	2	1A
*NNMT*	Nicotinamide N-methyltransferase	11	1 C
*NOS3*	Nitric oxide synthase 3 (endothelial cell)	13	5
*PADI4*	peptidyl arginine deiminase, type IV	28	1 C
*PAX3*	Paired box gene 3 (Waardenburg syndrome 1)	51	3
*PCMT1*	Protein-L-isoaspartate (D-aspartate) O-methyltransferase	7	1 C
*PDGFRA*	Platelet-derived growth factor receptor alpha	17	4
*PEMT*	phosphatidylethanolamine N-methyltransferase	14	1 C
*PGM2*	pgm2 methionine synthase interacting protein	19	1B
*PRMT8*	Protein arginine methyltransferase 8	54	1 C
*RAI1*	Retinoic acid induced 1	13	4
*SHMT1*	Serine hydroxymethyl transferase (C)	12	1A
*SHMT2*	Serine hydroxymethyl transferase (M)	4	1A
*SLC19A1*	Reduced folate carrier	4	2A
*T (Brachyury)*	T, brachyury homolog (mouse)	20	4
*TCblR*	Transcobalamin II receptor	5	2B
*TCN1*	Transcobalamin I	2	2B
*TCN2*	Transcobalamin II	20	2B
*TFAP2A*	Transcription factor AP-2 alpha (activating enhancer binding protein 2 alpha)	16	3
*TK1*	Thymidine kinase 1, soluble	12	1A
*TP53*	Tumor protein 53 (Li-Fraumeni syndrombe)	8	3
*TYMS*	Thymidylate synthase	4	1A

^*^Category Key: *1A*, Enzymatic (folate); *1B*, Enzymatic (B12); *1 C*, Enzymatic (methylation); *2A*, Transport (folate); *2B*, Transport (B12); 3, Transcription; 4, Development; 5, Other.

**Table 2:** SNPs Selected for Study in the Combined NTD Cohort

**RSID**	**Gene**	**Case–control Logistic Regression p-value**	**TDT p-value**	**Mother-Control Logistic Regression p-value**	**Log-Linear Maternal Genotype p-value**
rs2428735	*PADI4*	**0.0006**	0.2801	**0.0229**	0.4043
rs942459	*PADI4*	**0.0089**	0.3841	0.1002	.^*^
rs12743862	*ARID1A*	0.1030	**0.0314**	0.9124	.
rs12729444	*ARID1A*	0.1068	**0.0314**	0.9124	.
rs12726081	*ARID1A*	0.1030	**0.0314**	0.9124	.
rs12726287	*ARID1A*	0.1030	**0.0314**	0.9276	.
rs11247593	*ARID1A*	**0.0208**	0.1294	0.1815	0.1383
rs11247594	*ARID1A*	**0.0257**	0.1580	0.2126	0.1331
rs12752833	*ARID1A*	0.1030	**0.0314**	0.9124	.
rs12735646	*ARID1A*	0.1030	**0.0290**	0.9124	.
rs12737946	*ARID1A*	0.1030	**0.0314**	0.9124	.
rs12731749	*ARID1A*	0.0862	**0.0201**	0.8813	.
rs11247596	*ARID1A*	0.7022	**0.0311**	0.9123	0.9708
rs12733999	*CTH*	**0.0277**	1.0000	**0.0077**	.
rs10889869	*CTH*	0.9408	**0.0436**	0.2582	0.9918
rs12723350	*CTH*	**0.0472**	0.6803	**0.0120**	0.0572
rs16834388	*MTR*	0.1070	**0.0225**	0.9177	0.8117
rs10733117	*MTR*	0.1604	**0.0207**	0.5363	0.1956
rs10925238	*MTR*	0.0503	**0.0139**	0.9512	0.5724
rs4077829	*MTR*	0.0921	**0.0170**	0.7931	0.6494
rs12060570	*MTR*	0.0602	**0.0201**	0.9373	0.6843
rs2789352	*MTR*	0.2535	**0.0348**	0.7505	0.1245
rs7367859	*MTR*	0.2355	**0.0444**	0.5437	0.0571
rs10925260	*MTR*	0.1262	**0.0328**	0.8485	0.1713
rs2853522	*MTR*	0.1080	**0.0449**	0.8826	0.1600
rs2853523	*MTR*	0.2127	**0.0418**	0.7770	0.0594
rs3944004	*LRP2*	0.3963	**0.0048**	0.5667	0.2170
rs4667593	*LRP2*	0.7913	**0.0087**	0.3028	0.7203
rs16856530	*LRP2*	0.1458	0.4352	**0.0396**	0.7874
rs2024481	*LRP2*	0.9568	**0.0087**	0.2889	0.7601
rs10490131	*LRP2*	0.4348	**0.0140**	0.8093	0.8201
rs4668123	*LRP2*	0.1564	0.9379	**0.0487**	0.9494
rs2268373	*LRP2*	**0.0338**	**0.0229**	0.0718	0.4172
rs11886219	*LRP2*	**0.0491**	0.3924	**0.0034**	.
rs2268365	*LRP2*	0.6858	**0.0312**	0.6914	0.8866
rs2673164	*LRP2*	0.5967	0.7656	0.4009	**0.0231**
rs700550	*LRP2*	0.4767	0.2507	0.7529	**0.0383**
rs853988	*LRP2*	0.8386	0.6872	0.2807	**0.0048**
rs2673177	*LRP2*	**0.0095**	0.5791	**0.0015**	**0.0279**
rs10199676	*LRP2*	**0.0075**	0.7290	**0.0213**	0.9036
rs2389557	*LRP2*	**0.0336**	0.6507	0.1005	0.4636
rs16856843	*LRP2*	**0.0075**	0.9055	**0.0133**	0.9035
rs10806845	*T (Brachyury)*	**0.0399**	0.6331	**0.0010**	0.3100
rs3127441	*T (Brachyury)*	**0.0485**	0.6215	**0.0043**	0.8873
rs12200529	*T (Brachyury)*	0.5867	0.7758	**0.0278**	0.2407
rs16898752	*T (Brachyury)*	0.1443	1.0000	**0.0412**	0.2063
rs7753771	*T (Brachyury)*	0.5378	1.0000	**0.0384**	.
rs1001978	*T (Brachyury)*	0.5378	1.0000	**0.0384**	.
rs3099280	*T (Brachyury)*	0.9972	0.5149	**0.0199**	0.1406
rs4512347	*MFTC*	**0.0344**	0.6473	0.2789	.
rs10112450	*MFTC*	0.1575	**0.0196**	0.4726	0.3474
rs17803441	*MFTC*	**0.0016**	**0.0115**	0.2911	0.2838
rs3134260	*MFTC*	**0.0016**	**0.0086**	0.2513	0.2919
rs4876902	*EHMT1*	0.4872	0.0595	0.6710	0.9049
rs4526432	*EHMT1*	**0.0433**	**0.0251**	0.5323	0.4040
rs10752062	*CUBN*	0.2564	0.8216	**0.0254**	0.1471
rs17139378	*CUBN*	0.2113	0.6780	**0.0086**	0.1051
rs7100290	*CUBN*	0.4039	**0.0269**	0.6692	.
rs1276720	*CUBN*	0.2147	**0.0422**	0.5445	0.0714
rs1276721	*CUBN*	0.1737	**0.0206**	0.5381	0.2471
rs11254284	*CUBN*	0.4198	0.7175	**0.0474**	0.0548
rs11254313	*CUBN*	0.4354	0.4206	**0.0277**	0.3041
rs12258009	*CUBN*	0.7700	0.1582	0.6214	0.7119
rs17431655	*CUBN*	0.1453	0.8474	**0.0459**	.
rs17139663	*CUBN*	**0.0407**	0.8864	0.0579	0.5552
rs11254339	*CUBN*	**0.0490**	0.7928	0.0838	0.8621
rs1801228	*CUBN*	0.1118	0.8474	**0.0356**	.
rs12254816	*CUBN*	0.5285	0.6670	**0.0407**	0.1765
rs7899751	*CUBN*	0.4747	0.8386	**0.0433**	0.1183
rs11254375	*CUBN*	0.7142	0.6682	0.5854	**0.0332**
rs7070148	*CUBN*	0.1447	0.9013	**0.0029**	0.2028
rs2273737	*CUBN*	0.1136	0.9042	**0.0064**	0.3337
rs7096079	*CUBN*	**0.0202**	0.8897	**0.0432**	0.3447
rs2073588	*FTH1*	0.2129	**0.0046**	0.9390	.
rs17156616	*FTH1*	0.5455	**0.0127**	0.8668	.
rs195445	*FTH1*	0.9840	0.3738	0.8436	0.7908
rs7957531	*LRP6*	0.4835	0.9068	**0.0407**	0.2288
rs10845493	*LRP6*	0.1614	**0.0149**	0.9106	0.7491
rs17302049	*LRP6*	0.4464	**0.0242**	0.8904	0.9593
rs1181334	*LRP6*	0.2935	**0.0211**	0.9466	0.9000
rs12309338	*LRP6*	0.7156	0.6056	**0.0329**	.
rs3760183	*PEMT*	**0.0146**	**0.0111**	0.2960	.
rs16961845	*PEMT*	0.2442	0.8788	**0.0043**	.
rs4646342	*PEMT*	0.4687	0.6714	0.7126	**0.0351**
rs2350631	*PEMT*	0.0641	0.6803	**0.0181**	0.3410
rs9910090	*SHMT1*	**0.0342**	0.1508	0.1370	0.7042
rs9901160	*SHMT1*	0.5153	**0.0153**	0.9278	0.4380
rs4911263	*DNMT3B*	0.0686	0.8332	**0.0145**	**0.0475**
rs6058896	*DNMT3B*	**0.0460**	**0.0016**	0.5438	.
rs447833	*ADA*	0.0832	**0.0090**	0.3777	0.6497
rs2299686	*ADA*	0.4258	0.7269	**0.0391**	0.2794
rs427483	*ADA*	0.3645	0.5276	**0.0275**	0.5681
rs6094017	*ADA*	0.1869	0.5169	**0.0088**	0.6004

^*^ (.) = Failure to converge (no result).

### Combined analyses

**Figure 2:**
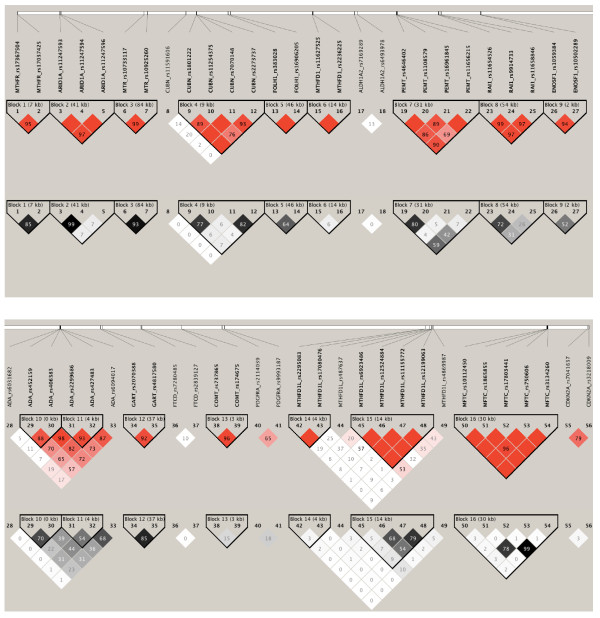
**Linkage Disequilibrium Relationships Between SNPs Significantly Associated with NTDs.** Sixty-eight SNPs were associated with case or maternal effects (p < 0.01, uncorrected). Of these, 56 SNPs occur in the same gene as at least one other significantly associated SNP. Their D’ (red scale) and r^2^ (gray scale) relationships are shown. These SNPs are plotted by relative position. Haplotype blocks defined by a Solid Spine of LD (D’) are outlined in black. Each SNP is numbered and the gene and dbSNP accession number for each are: 1) MTHFR_rs17367504; 2) MTHFR_rs17037425; 3) ARID1A_rs11247593; 4) ARID1A_rs11247594; 5) ARID1A_rs11247596; 6) MTR_rs10733117; 7) MTR_rs10925260; 8) CUBN_rs11591606; 9) CUBN_rs1801222; 10) CUBN_rs11254375; 11) CUBN_rs7070148; 12) CUBN_rs2273737; 13) FOLH1_rs383028; 14) FOLH1_rs16906205; 15) MTHFD1_rs11627525; 16) MTHFD1_rs2236225; 17) ALDH1A2_rs7169289; 18) ALDH1A2_rs6493978; 19) PEMT_rs4646402; 20) PEMT_rs1108579; 21) PEMT_rs16961845; 22) PEMT_rs11656215; 23) RAI1_rs11654526; 24) RAI1_rs9914733; 25) RAI1_rs11658846; 26) ENOSF1_rs1059384; 27) ENOSF1_rs10502289; 28) ADA_rs6031682; 29) ADA_rs452159; 30) ADA_rs406383; 31) ADA_rs2299686; 32) ADA_rs427483; 33) ADA_rs6094017; 34) GART_rs2070388; 35) GART_rs4817580; 36) FTCD_rs7280485; 37) FTCD_rs2839127; 38) COMT_rs737865; 39) COMT_rs174675; 40) PDGFRA_rs2114039; 41) PDGFRA_rs9993187; 42) MTHFD1L_rs2295083; 43) MTHFD1L_rs17080476; 44) MTHFD1L_rs487637; 45) MTHFD1L_rs6923486; 46) MTHFD1L_rs12524884; 47) MTHFD1L_rs11155772; 48) MTHFD1L_rs12199063; 49) MTHFD1L_rs4869987; 50) MFTC_rs10112450; 51) MFTC_rs1865855; 52) MFTC_rs17803441; 53) MFTC_rs750606; 54) MFTC_rs3134260; 55) CDKN2A_rs7041637; 56) CDKN2A_rs3218009

In the combined analyses each SNP was evaluated for contributing to NTD risk by twelve association tests: case and maternal effects were each evaluated using three case–control (or mother-control) tests and three family-based tests (see Methods). There were 68 SNPs in 30 genes that showed an association at the p < 0.01 level by any of the twelve tests (Table 3). Of these, twelve genes contained a single associated SNP. The remaining 56 SNPs were found in 18 genes. Not all associations were independent. Areas of interest were covered by tagSNPs as well as additional SNPs for physical coverage; as a result there were seven SNP pairs in this set with a linkage disequilibrium (LD) relationship above the threshold (r^2^ ≥ 0.8) selected for tagging in this study (Figure 2, *ARID1A*_rs11247593 and *ARID1A*_rs11247594, *CUBN*_rs7070148 and *CUBN*_rs2273737, *GART*_rs2070388 and *GART*_rs4817580, *MFTC*_rs17803441 and *MFTC*_rs3134260, *MTHFR*_rs17367504 and *MTHFR*_rs17037425, *MTR*_rs10733117 and *MTR*_rs10925260, and *PEMT*_rs4646402 and *PEMT* rs1108579). Notably, for many genes, the associated SNPs occur in the same haplotype block (solid spine of LD based on D’ relationships), implying a single association signal for that gene (Figure 2, *GART, COMT, MFTC, MTHFR, ARID1A, MTR, FOLH1, MTHFD1, PEMT, RAI1, ENOSF1*). A single signal probably also accounts for the significant SNP pairs seen in *PDGFRA* (D’ = 0.65) and *CDKN2A* (D’ = 0.79). Genes exhibiting more than one independent SNP association include *FTCD* (D’ = 0.10), *MTHFD1L* (two separate haplotype blocks and two SNPs, D’ ≤ 0.57), *CUBN* (one haplotype block and a SNP, D’ ≤ 0.20), *ALDH1A2* (D’ = 0.13) and *ADA* (a weak haplotype block and a SNP, D’ ≤ 0.19).

**Table 3:** p-values for Evaluated SNPs Showing Association (p < 0.01) with NTDs

**Gene/SNP**	**TDT**	**LL CC Rec**	**LL CC Dom**	**LR CC Cont**	**LR CC Rec**	**LR CC Dom**	**LL Mat 2DOF**	**LL Mat Rec**	**LL Mat Dom**	**LR MC Cont**	**LR MC Rec**	**LR MC Dom**
*MFTC rs17803441	0.0300	0.1456	**0.0098**	**0.0003**	**0.0013**	0.0146	0.4634	0.6352	0.2195	0.1928	0.2373	0.3535
*CDKN2A* rs3218009	0.1284	0.9996	0.1786	0.0426	0.9775	0.1009	.^**^	0.2734	**0.0004**	0.3032	0.5724	0.3296
*ADA rs2299686	0.8288	0.7819	0.5289	0.2994	0.3991	0.3958	0.2165	0.1923	0.1183	**0.0010**	0.0479	**0.0005**
*PEMT rs4646402	**0.0009**	0.1861	**0.0005**	0.1374	0.3728	0.1348	0.7219	0.5709	0.7630	0.7482	0.5319	0.9693
*MFTC rs3134260	0.0225	0.1301	**0.0059**	**0.0006**	**0.0021**	0.0141	0.4213	0.4460	0.2195	0.2102	0.2594	0.3484
*PEMT rs1108579	**0.0006**	0.0463	**0.0014**	0.0462	0.0482	0.1846	0.7076	0.4098	0.8059	0.9490	0.9274	0.9873
*PEMT rs11656215	**0.0053**	**0.0008**	0.3818	0.2070	0.1461	0.5412	0.7689	0.5639	0.8703	0.8211	0.3273	0.5520
*CUBN rs7070148	0.4864	0.4012	0.6033	0.0909	0.8065	0.0746	0.4458	0.4235	0.2551	**0.0010**	0.1735	**0.0013**
*ADA rs406383	0.0590	0.0311	0.3102	0.8420	0.7244	0.6858	0.1205	0.4468	0.0398	**0.0012**	**0.0021**	0.0136
*GART rs2070388	**0.0012**	0.5668	**0.0012**	0.0724	0.1415	0.1209	0.4526	0.2734	0.4332	0.5831	0.1515	0.8478
*ADA rs6031682	0.5166	0.6123	0.6101	**0.0052**	0.9578	**0.0016**	0.8837	0.8085	0.6321	**0.0048**	0.8196	**0.0019**
*ADA rs427483	0.6597	0.8842	0.3286	0.2509	0.1948	0.7039	0.4568	0.3370	0.2699	**0.0018**	0.0137	**0.0063**
*DNMT3A* rs7560488	0.1538	**0.0021**	0.6359	0.8325	0.8348	0.8823	0.5544	0.3633	0.3848	0.7779	0.9764	0.6509
*CUBN rs2273737	0.5067	0.7062	0.5487	0.0780	0.3584	0.0936	0.4034	0.4843	0.1971	**0.0021**	0.2034	**0.0026**
*MTHFD1* rs2236225	0.1505	0.9554	0.0257	0.2570	0.3372	0.3869	0.8915	0.6549	1.0000	0.0139	0.2593	**0.0022**
*T rs10806845	0.2894	0.3889	0.4518	0.0830	0.0370	0.4302	0.2438	0.1204	0.2378	**0.0033**	**0.0023**	0.0701
*FOLH1* rs383028	0.2073	0.4975	0.2542	**0.0050**	0.3617	**0.0051**	0.1033	1.0000	0.0359	**0.0054**	0.9171	**0.0024**
*CUBN rs1801222	1.0000	0.5013	0.4017	0.1514	0.7600	0.0181	0.2822	0.1682	0.2448	**0.0091**	0.1439	**0.0024**
*ADA rs6094017	0.2199	0.6211	0.2009	0.3960	0.8060	0.3126	0.7449	0.5105	0.5466	**0.0026**	0.0479	**0.0046**
*ARID1A rs11247593	0.0154	0.0502	0.0747	**0.0027**	**0.0090**	0.0265	0.1455	0.4060	0.1491	0.0615	0.0458	0.5659
*COMT* rs174675	0.0795	0.7444	**0.0028**	0.8722	0.5266	0.4898	0.9321	0.7778	0.7457	0.3665	0.2494	0.9574
*ENOSF1 rs1059384	0.6886	0.3493	0.9141	0.0333	**0.0028**	0.2734	0.9279	0.8084	0.8226	0.0848	**0.0063**	0.4655
*GART rs4817580	**0.0029**	0.9996	**0.0042**	0.0849	0.9745	0.1174	.	0.9996	0.9223	0.4818	0.9747	0.6024
*DNMT3B rs6058896	**0.0029**	**0.0052**	.	0.0167	0.0244	0.9744	.	0.2022	.	0.0905	0.1265	0.9744
*MTHFD1L* rs12199063	0.2857	0.3198	0.5830	0.1248	0.3647	0.0575	0.0855	0.0437	0.7457	**0.0035**	**0.0029**	0.1898
*MTHFD1L* rs6923486	0.1738	0.3458	0.1249	0.1348	0.1283	0.5396	0.2973	0.2651	0.1785	**0.0030**	**0.0090**	0.0348
*COMT* rs737865	0.0112	0.0149	0.1106	0.5284	0.3462	0.2354	0.1983	0.1326	0.1587	0.0852	**0.0031**	0.5815
*MTR rs10925260	0.0290	**0.0031**	0.9246	0.6329	0.4355	0.8813	0.4513	0.7071	0.2073	0.1865	0.2408	0.3423
*ADA rs452159	0.0577	0.5282	**0.0047**	0.7260	0.9583	0.4298	0.1675	0.0627	0.3633	**0.0059**	0.0578	**0.0032**
*TFAP2A* rs17635655	0.8108	0.6367	0.7196	0.0116	0.0236	0.0813	0.1688	0.0876	0.8527	**0.0037**	**0.0075**	0.0645
*CUBN* rs11591606	0.1808	0.2426	0.4253	0.4459	0.6193	0.2639	0.8403	0.6321	0.6380	0.3280	0.9827	**0.0058**
*MTHFD1L* rs2295083	0.6044	0.2928	0.2330	0.0168	**0.0038**	0.7292	0.2734	0.1752	0.5943	0.0558	0.0342	0.7136
*PEMT rs16961845	0.5691	0.4101	0.2719	0.4982	0.4864	0.9363	.	**0.0082**	.	**0.0072**	**0.0048**	0.9255
*ALDH1A2 rs7169289	0.1063	0.6803	0.0967	0.0214	0.5178	**0.0043**	0.4346	0.2067	0.6266	0.0633	0.6737	0.0513
*MTR rs10733117	**0.0099**	0.4520	**0.0043**	0.6020	0.7559	0.6099	0.2998	0.1791	0.2388	0.1436	0.3788	0.1542
*MTHFD1L* rs17080476	0.0233	0.9996	0.0949	**0.0070**	0.0244	0.0320	.	0.5299	0.9136	0.1357	0.0994	0.2676
*MAT2B* rs17535909	0.0102	**0.0045**	0.5083	0.2745	0.1949	0.7860	0.2061	0.3321	0.2406	0.8030	0.6685	0.8863
*GNMT* rs9462856	0.0601	0.9187	**0.0048**	0.7463	0.7773	0.3914	0.8419	0.5788	1.0000	0.6497	0.8424	0.5744
*CTH rs12733999	0.1546	0.1603	0.7406	**0.0051**	**0.0048**	0.6384	.	0.1477	0.3414	0.0167	0.0285	0.1264
*CDKN2A* rs7041637	0.4033	0.0881	0.3011	**0.0048**	**0.0067**	0.1269	0.3323	0.2793	0.1978	0.1369	0.3445	0.0716
*RAI1 rs9914733	0.2215	0.1984	0.4266	0.8834	0.4610	0.6300	0.0980	0.6952	0.0320	0.0133	0.8111	**0.0049**
*FTCD rs2839127	0.2296	0.0301	0.7043	0.8551	0.2725	0.8462	0.0195	0.2266	**0.0053**	0.1576	0.0483	0.3997
*PDGFRA* rs9993187	0.3615	0.0802	0.7374	0.0639	0.1576	0.0123	0.4850	0.4235	0.2999	**0.0083**	0.8603	**0.0054**
*MTHFD1L* rs4869987	0.1603	0.6351	0.0458	0.1693	0.2368	0.2939	0.0284	0.3579	0.0511	0.0167	0.1767	**0.0061**
*ARID1A rs11247594	0.0376	0.0989	0.1141	**0.0059**	0.0159	0.0438	0.2152	0.2972	0.3060	0.0758	0.0601	0.5558
*ARID1A rs11247596	**0.0059**	0.1178	0.0151	0.0570	0.2900	0.0682	0.6110	0.3713	0.8781	0.5223	0.0978	0.9777
*FOLH1* rs16906205	0.2509	0.5003	0.3078	0.0993	0.9856	0.0730	.	0.9996	**0.0060**	0.0160	0.9856	0.0102
*FTCD rs7280485	0.5455	1.0000	0.2928	0.9719	0.9612	1.0000	0.0402	0.2205	0.0796	0.1847	0.9315	**0.0062**
*ENOSF1* rs10502289	0.4050	0.0606	0.9176	0.2608	**0.0084**	0.8743	0.9012	0.6553	0.8231	0.7398	0.4196	0.9254
*MFTC rs10112450	**0.0065**	0.0154	0.1380	0.1077	0.0765	0.7454	0.4967	0.2680	0.4937	0.5459	0.7307	0.3836
*MFTC rs1865855	0.0339	0.2226	0.0596	**0.0066**	0.2347	**0.0091**	0.4858	0.3270	0.3615	0.3489	0.3248	0.2103
*MTHFD1* rs11627525	0.7010	0.9468	.	0.7285	0.5723	0.9802	.	**0.0067**	1.0000	0.5750	0.9194	0.1216
*GGH* rs11988534	0.0409	**0.0067**	0.6955	0.2427	0.1156	0.8060	0.6044	0.6257	0.4665	0.9112	0.8775	0.5796
*MTHFR* rs17037425	1.0000	0.8419	0.6233	0.4158	0.2803	0.6769	0.0149	**0.0068**	0.7817	0.1828	0.0796	0.4756
*MTHFD1L* rs487637	0.1946	0.4121	0.0364	0.0162	0.5831	**0.0069**	0.8861	0.7152	0.8488	0.2125	0.5912	0.2003
*DMGDH* rs663646	0.2749	**0.0070**	0.8907	0.7786	0.1838	0.7429	0.8537	0.5782	0.9263	0.5080	0.1877	0.8789
*NNMT* rs10400393	**0.0074**	0.1090	0.0203	0.8395	0.6189	0.9926	0.9369	0.7317	1.0000	0.5062	0.4840	0.6355
*CUBN rs11254375	0.3772	0.5709	0.4612	0.6008	0.1449	0.7839	0.0331	0.6321	**0.0097**	0.0274	**0.0074**	0.2404
*MTHFD1L* rs11155772	0.1876	0.1492	0.8084	0.5140	0.8021	0.2358	0.2306	0.0932	1.0000	0.0126	**0.0075**	0.5404
*ALDH1A2* rs6493978	0.5450	0.0984	0.5323	0.1136	0.6939	0.0308	0.9785	0.9093	0.8399	0.0333	0.4523	**0.0076**
*RAI1* rs11658846	0.2504	1.0000	0.2329	0.1382	0.6534	0.1409	0.3954	0.9994	0.1351	0.0104	0.6431	**0.0088**
*LRP2* rs3914468	0.8840	0.1061	0.1878	0.2044	0.0181	0.7696	0.1386	0.7577	0.0734	0.2555	**0.0080**	0.9562
*PDGFRA* rs2114039	**0.0081**	0.1297	0.0185	0.9479	1.0000	0.9317	0.7483	0.4513	0.9146	0.5652	0.7360	0.5786
*MTHFR* rs17367504	0.7868	0.3302	0.9193	0.7251	0.6814	0.5857	0.0174	0.7817	**0.0081**	0.2908	0.4835	0.1536
*EHMT1 rs7039441	0.4612	0.3106	0.8211	0.9611	0.8037	0.9150	0.0319	0.2588	**0.0090**	0.0981	0.8608	0.0282
*MFTC rs750606	0.0378	0.7460	0.0220	**0.0091**	0.3426	**0.0090**	0.2301	0.0994	0.3619	0.3230	0.3719	0.1578
*MTHFD1L* rs12524884	0.6841	0.7842	0.6630	0.2910	0.5126	0.1146	0.2320	0.0876	0.7633	**0.0094**	0.0142	0.1789
*RAI1 rs11654526	0.1373	0.0904	0.3348	0.9082	0.6677	0.7709	0.1313	0.4417	0.0450	**0.0099**	0.2692	0.0106

^*^ Indicates SNP was genotyped in the full cohort (570 triads, 999 controls). Otherwise SNP was genotyped in the primary sample set (320 triads, 341 controls).

^**^ (.) = Failure to converge (no result).

Note: *MTHFD1* rs2236225 and all *MTHFD1L* SNPs in this table were previously examined for NTD association in this dataset [23,25,27].

We ranked SNPs by the lowest p-value for any test, accounting for relatedness to other highly significant SNPs. The nine genes exhibiting the 10 strongest association signals were: *MFTC, CDKN2A, ADA, PEMT, CUBN, GART, ADA, DNMT3A, MTHFD1* and *T (Brachyury)* (Table 4). *MFTC, ADA, PEMT* and *CUBN* contained more than one significant SNP, and *ADA* showed evidence of two independent association signals.

**Table 4:** The Ten Independent^*^Association Signals Exhibiting the Lowest Uncorrected p-values for Any Test of NTD Association

	**Gene/SNP**	Risk Allele Frequency^**^	Cohort***	**Model**	**OR**	**95% CI (LL)**	**95% CI (UL)**	**P**
1 *MFTC*								
rs17803441		0.07	Combined	LR CC Continuous	1.61	1.23	2.08	0.0003
*MFTC*								
rs3134260		0.07	Combined	LR CC Continuous	1.56	1.22	2.04	0.0006
2 *CDKN2A*				LL Maternal effect,				
rs3218009		0.13	Primary	Dominant	2.32	1.45	3.71	0.0004
3 *ADA*								
rs2299686		0.45	Combined	LR MC Dominant	1.56	1.22	2.04	0.0005
*ADA*								
rs406383		0.25	Combined	LR MC Continuous		1.12	1.58	
*ADA*								
rs427483		0.33	Combined	LR MC Continuous	1.28	1.10	1.52	0.0018
4 *PEMT*								
rs1108579		0.52	Combined	TDT	1.47	1.18	1.82	0.0006
*PEMT*								
rs11656215		0.49	Combined	LL Case effect, recessive	1.68	1.24	2.28	0.0008
*PEMT*								
rs4646402		0.57	Combined	LL Case effect, dominant	1.67	1.25	2.17	0.0005
5 *CUBN*								
rs7070148		0.90	Combined	LR MC Continuous	1.64	1.22	2.17	0.0010
*CUBN*								
rs2273737		0.89	Combined	LR MC Continuous	1.54	1.18	2.04	0.0021
6 *GART*								
rs2070388		0.91	Combined	TDT	1.89	1.28	2.78	0.0012
*GART*								
rs2070388		0.91	Combined	LL Case effect, dominant	1.96	1.30	2.94	0.0012
7 *ADA* rs6031682		0.16	Combined	LR CC Dominant	1.49	1.16	1.92	0.0016
8 *DNMT3A* rs7560488		0.42	Primary	LL Case effect, recessive	2.10	1.31	3.38	0.0021
9 *MTHFD1* rs2236225		0.40	Primary	LR MC Dominant	1.96	1.28	3.03	0.0022
10 *T (Brachyury)* rs10806845		0.51	Combined	LR MC Recessive	1.54	1.16	2.00	0.0023

^*^ LD between SNPs within an association signal are as follows: *MFTC* (D’ = 1, r^2^ = 0.993), *ADA* (D’ > 0.822, r^2^ > 0.394), *PEMT* (D’ > 0.693, r^2^ > 0.428) and *CUBN* (D’ = 0.935, r^2^ = 0.823). *ADA* rs6031682 was designated as an independent association signal because it shares very little LD (D’ < 0.206, r^2^ < 0.016) with the other *ADA* SNPs (rs2299686, rs406383, rs427483).

^**^Frequency of the risk allele was calculated using control sample genotypes.

^***^ Primary: 320 triads, 341 controls. Combined: 570 triads, 999 controls.

SNPs in *MFTC, PEMT* and *ADA* account for seven of the top ten SNPs (Table 4). In *MFTC*, these two SNPs (rs17803441 and rs3134260) are essentially in perfect LD (D’ = 1.0, r^2^ = 0.99). As expected, they yielded very similar evidence of NTD risk in a continuous model of logistic regression (rs17803441: OR = 1.61 [1.23-2.08], p = 0.0003, Risk Allele Frequency (RAF) = 0.07; rs3134260: OR = 1.56 [1.22-2.04], p = 0.0006, RAF = 0.07), as well as a recessive model of logistic regression (rs17803441: OR = 1.59 [1.19-2.08], p = 0.0013; rs3134260: OR = 1.54 [1.18-2.04], p = 0.0021). There are a total of five highly significant (p < 0.01) *MFTC* SNPs and they are all consistent with a case effect. *MFTC* rs10112450 is significant by the transmission disequilibrium test (TDT, GRR = 1.42, p = 0.0065, RAF = 0.79), and the remaining *MFTC* SNPs (rs1865855 and rs750606) show evidence of NTD risk to the case by logistic regression with a continuous or dominant model (p < 0.0091). Because all five of these SNPs fall in the same haplotype block (D’ > 0.96), it is likely there is a single variant in this region responsible for the association signals.

Three SNPs in *PEMT* were in the top ten SNPs, and two of these are in high linkage disequilibrium (rs1108579 and rs4646402; D’ = 1.0, r^2^ = 0.809). These SNPs also yield very similar evidence of NTD risk by TDT (rs1108579: GRR = 1.47, p = 0.0006, RAF = 0.52; rs4646402: GRR = 1.43, p = 0.0009, RAF = 0.57). *PEMT* rs11656215 is less strongly linked to these SNPs (r^2^ < 0.6), and shows association with NTD risk by TDT (GRR = 1.35, p = 0.0053, RAF = 0.49) and by log-linear analysis of a recessive model (GRR = 1.68 [1.24-2.28], p = 0.0008). A fourth highly significant SNP was found in the maternal analysis (rs16961845). It shows evidence of maternal risk in a recessive model of log-linear analysis (GRR = 1.92 [1.18-3.11], p = 0.0082), as well as recessive (p < 0.0048) and continuous (p < 0.0072) models of logistic regression. By r^2^ measures of LD, this SNP is the least related to the other three SNPs (r^2^ < 0.08), although all four of these SNPs fall in the same haplotype block (Figure 2).

Lastly, three SNPs in *ADA* were among the ten SNPs with the lowest p values. They show evidence of LD by D’ (≥0.82) but less so by r^2^ (≤0.54). These three SNPs are all significantly associated with a maternal effect by logistic regression of a continuous model (rs2299686: OR = 1.30 [1.11-1.52], p = 0.0010, RAF = 0.45; rs427483: OR = 1.28 [1.10-1.52], p = 0.0018, RAF = 0.33; rs406383: OR = 1.33 [1.12-1.58], p = 0.0012, RAF = 0.25). Three other SNPs in *ADA* (rs6031682, rs452159 and rs6094017) also showed a highly significant association with a maternal effect in a continuous model of logistic regression (p < 0.0059). All six SNPs were highly significant (p < 0.01) for association with maternal risk in either a recessive model (rs406383, p = 0.0021) or dominant model (the other five SNPs, p ≤ 0.0063) of logistic regression. Additionally, *ADA* rs6031682 shows association with case risk in a continuous model of logistic regression (1.38 [1.10-1.73], p = 0.0052, RAF = 0.84). These six SNPs do not appear to be strongly linked; no haplotype blocks (solid spine of LD) larger than two SNPs were identified. It appears that rs6031682 is clearly outside of a degraded haplotype block consisting of the other five SNPs.

*CUBN* was the only other gene with more than one SNP found in the ten most strongly associated signals. *CUBN* rs7070148 and *CUBN* rs2273737 were both significant for association with maternal risk in a continuous model of logistic regression (OR = 1.64 [1.22-2.17], p = 0.0010, RAF = 0.90 and OR = 1.54 [1.18-2.04], p = 0.0021, RAF = 0.89, respectively). These SNPs are highly linked (D’ = 0.935, r^2^ = 0.823). There are three other highly significant (p < 0.01) SNPs in *CUBN*. Two other SNPs in the same haplotype block with *CUBN* rs7070148 and *CUBN* rs2273737 (rs1801222 and rs11254375, D’ < 0.76) also showed an association with maternal effects. A third SNP, *CUBN* rs11591606, is outside this block (D’ < 0.20) and is associated with maternal risk in a dominant model of logistic regression (OR = 4.15 [1.51-11.39], p = 0.0058, RAF = 0.17).

Correction for multiple testing was performed for three of the twelve tests of association. No adjusted p-value was found to remain significant, and no further correction was performed.

## Discussion

This study represents a new scale of evaluation of genetic contribution to NTD risk. Common variants in 82 biologically plausible candidate genes were tested for association with NTDs in a large Irish population. Seventeen variants in nine genes account for the ten most significant associations observed. *CDKN2A, GART, DNMT3A, MTHFD1* and *T (Brachyury)* contained a single SNP among the ten lowest p-values observed for all tests. In contrast, *MFTC, ADA, PEMT*, and *CUBN* each contained more than one such SNP. This seems to be due to strong LD relationships between the associated SNPs. The only exception is in *ADA*, which shows evidence of two strong, unrelated association signals.

*ADA* (adenosine deaminase) converts adenosine to inosine by removal of an amino group. Deficiency in this enzyme causes severe combined immunodeficiency disease (SCID), which is characterized by compromise of both T cells and B cells. Interestingly, *ADA* activity was significantly elevated in a study of 68 pregnant women carrying a fetus with a central nervous system malformation [39]; of these women, 17 had a spina bifida pregnancy. Consistent with this, six unrelated (r^2^ < 0.70), noncoding *ADA* SNPs were found in the current study to be associated with maternal risk of carrying an NTD pregnancy (p ≤ 0.006, uncorrected). Genetic variation in *ADA* may contribute to maternal risk of NTDs. In addition, this gene was the only one to exhibit two independent association signals among the top ten signals observed. This may indicate that there is more than one allele associated with risk or the same allele has recurred on more than one haplotype. *ADA* rs6031682 shows evidence of case effects (p = 0.0016) as well as maternal effects (p = 0.0019) in log-linear analyses of a dominant model, and it is clearly independent of the other significant *ADA* SNPs (D’ < 0.19). It would be of interest to test the associated *ADA* SNPs in an independent study, especially since the scale of correction would be much smaller in a focused study.

*PEMT* (phosphatidylethanolamine N-methyltransferase) plays a role in choline metabolism. It converts phosphatidylethanolamine to phosphatidylcholine in the liver; phosphatidylcholine is a major component of cell membranes. This role for choline can compete with its role as a methyl donor. Choline can be converted to betaine, which acts as a methyl donor in an alternate, folate-independent conversion of homocysteine to methionine. This link between folate and choline metabolism makes *PEMT* an interesting candidate gene, and interactions between *PEMT* SNPs have been reported to be associated with NTDs. In a case–control study, single SNP effects were not observed, although some compound genotypes for *PEMT* rs7946 and *PEMT* rs897453 were associated with decreased NTD risk [40]. The latter variant was not directly tested in the current study, and no association for *PEMT* rs7946 was observed in this Irish sample [37]. However, one related SNP pair (r^2^ = 0.80) and two other SNPs (r^2^ < 0.60) in *PEMT* falling in the same haplotype block (D’ ≥ 0.69) showed NTD association (p < 0.0053, uncorrected) in the current study, suggesting a role for this gene in NTD risk. Unlike the other three SNPs that exhibited case effects, the least related SNP in this block (*PEMT* rs16961845, 0.69 ≤ D’ ≤ 0.89) was positive for maternal effects by three tests of association. This intronic SNP is in strong r^2^ LD with 6 other intronic SNPs, making it difficult to speculate about its function. It is also difficult to discern whether the associated SNPs in this block represent independent signals for case risk and for maternal risk, or whether a single signal for a case effect is being detected. Therefore, further studies on the variation of this gene and NTDs are warranted.

*MFTC* (mitochondrial folate transporter/carrier, *SLC23A32*) transports folate from the cytoplasm into the mitochondria. Some folate metabolic reactions occur in both the cytoplasm and in mitochondria via compartment-specific enzymes. The mitochondrion produces the majority of the one carbon units used by the cell (reviewed in [41]). As the genes coding for these mitochondrial enzymes have been identified, they have been shown to be intriguing and relevant candidates for NTD studies. For example, we previously reported that the gene encoding 5, 10-methylene-tetrahydrofolate dehydrogenase 1-like (*MTHFD1L*) contains a polymorphism associated with NTDs [27]. Genetic variation affecting mitochondrial folate transport may also contribute to NTDs, as seen by our finding that 5 of 11 tested *MFTC* SNPs showed association (p < 0.01) with NTD risk in cases. This gene falls in a region of very high D’ LD; the haplotype block containing these five SNPs extends ~92 kb and contains two other genes: *DCAF13* (*DDB1* and *CUL4* associated factor 13) and *CTHRC1* (collagen triple helix repeat containing 1). Any SNP in this large haplotype block could be the causative variant driving the observed associations. As the only coding SNP in *MFTC*, the best candidate is rs17803441 (R117H). However, as arginine and histidine are both polar, basic amino acids, this is a fairly conservative change. We observed a minor allele frequency of 0.07 in this study. Conservation of the more common arginine residue is observed in chimp, wolf, cow, mouse, rat and zebrafish, but not in chicken or invertebrates. All of the SNPs in high LD (r^2^ > 0.7) with *MFTC* rs17803441 (R117H) in this block are intronic or intergenic. This SNP also had the lowest p-value for any test of association of all SNPs tested in this study. It would be of great interest to determine in an independent population whether it contributes to NTD risk.

*CUBN* encodes the intestinal receptor responsible for the uptake of the vitamin B12-intrinsic factor complex. It is also expressed in the kidney, where it is involved in reabsorption of many proteins and vitamins, including vitamin B12. This gene spans more than 300 kb of DNA. The only reported SNP association in *CUBN* is for rs1907362, which was associated with case risk in a Dutch population [35]. In contrast, we observed two highly significant SNPs in *CUBN* (rs7070148 and rs2273737) associated with maternal NTD risk. Due to their high LD these SNPs represent a single association signal. There were three other highly associated (p < 0.01) SNPs in this gene. *CUBN* rs11591606 was associated with maternal risk, and is in a smaller haplotype block at the 3’ end of the gene. Two other *CUBN* SNPs (rs1801222 [S253F] and rs11254375) were also highly associated (p < 0.01) with maternal risk and are in the same ~30 kb haplotype block with rs7070148 and rs2273737 at the 5’ end of the gene. While there are many SNPs in this block that could be the causal risk SNP, rs1801222 is of interest since it is a coding SNP (S253F) that was significantly associated with lower serum vitamin B12 levels in a meta-analysis of three genome wide association studies of three Caucasian populations [42]. This does not prove that *CUBN* rs1801222 is the causal SNP in either study, but it is consistent with the hypothesis that this SNP or another CUBN polymorphism linked to it within this haplotype block lowers vitamin B12 levels and thereby increases risk of an NTD pregnancy.

Multiple highly significant SNPs in *ADA, PEMT, MFTC* and *CUBN* account for half of the ten strongest association signals observed. The remaining five association signals are equally compelling. *MTHFD1* rs2236225 (R653Q) was previously reported as a maternal NTD risk factor in the current study population [23,25] and others [22,24], while the other four signals represent new associations. First, *CDKN2A* rs3218009 was associated with maternal risk for NTDs. *CDKN2a* is a tumor suppressor gene that codes for several isoforms, including *ARF* (alternate open reading frame), a protein that stabilizes p53. A subset of mice carrying p53 null alleles exhibit overgrowth of neural tissue, supporting the importance of this pathway in normal neural tube development. Second, the same highly significant p-value was obtained for *GART* rs2070388 by two tests for case effect: TDT and log-linear analysis of a dominant model of case effect. *GART* is a trifunctional enzyme (phosphoribosylglycinamide formyltransferase, phosphoribosylglycinamide synthetase, phosphoribosylaminoimidazole synthetase) involved in *de novo* purine synthesis. For its phosphoribosylglycinamide activity, *GART* uses N^10^-formyl tetrahydrofolate as a one-carbon donor in the synthesis pathway of inosine monophosphate (IMP), a purine precursor. Interestingly, *GART* rs4817579 in intron 2 has been associated with cleft lip and/or palate plus dental anomalies [43]. This variant was not tested in the current study, and the absence of *GART* rs2070388 from the HapMap data prevents us from evaluating the relatedness of these markers.

Third, *DNMT3A* rs7560488 was associated with NTD risk in cases. This gene encodes a DNA methyltransferase involved in de novo methylation during development. The folate pathway generates S-adenosyl methionine, which is used by *DNMT3A* as a methyl donor. Fourth, *T (Brachyury)* rs10806845 was associated with maternal NTD risk. The *T (Brachyury)* gene encodes a transcription factor involved in mesoderm formation and differentiation, and mice null for *T (Brachyury)* do not survive to term due to a number of developmental abnormalities, including fusion of the neural tube to the gut. Although previous studies differ in whether genetic variation in *T (Brachyury)* contributes to NTD risk in cases [44-48], our observation may be the first indication of its contribution to maternal risk of carrying an affected fetus.

Although no associations remained significant after conservative adjustment for multiple tests, it remains very possible that some of the evaluated candidates do in fact contribute to NTDs. The scale of our study design (using twelve tests of association to evaluate 1441 candidate SNPs) could contribute to Type II errors. This possibility is supported by the fact that of three SNPs previously reported to be associated with NTDs in this cohort (*MTHFR* 677 C > T [49,50], *MTHFD1* R653Q [23,25], *TCblR* G220R [34]) only one was observed to be associated in the current study design (Table 5). Only *MTHFD1* R653Q was found to be significantly associated in the primary phase of the analysis, which was performed on approximately half the samples. *MTHFR* 677 C > T and *TCblR* G220R were only found to be associated (p < 0.05, uncorrected) when the full cohort of samples were used. This suggests the possibility that the stringency of correction may be too high. Additionally, it is important to note that *MTHFD1* R653Q was ninth among the top ten association signals in this study (Table 4). This suggests that a number of the ten strongest association signals observed in this study play a role in NTD risk, and they should be high priority candidates for further study.

**Table 5:** Significant (Uncorrected) Analyses of SNPs Previously Reported as NTD Risk Factors in this Population

**Gene/SNP**	MAF^*^	Cohort^**^	**Model**	**OR**	**95% CI (LL)**	**95% CI (UL)**	**P**
*MTHFR* rs1801133 677 C > T	0.32	Combined	LR CC Continuous	1.23	1.05	1.45	0.011
		Combined	LR CC Recessive	1.26	1.01	1.57	0.041
		Combined	LR CC Dominant	1.44	1.04	2.00	0.029
		Combined	LR MC Continuous	1.22	1.04	1.43	0.017
		Combined	LR MC Recessive	1.25	1.01	1.57	0.045
							
*MTHFD1* rs2236225 R653Q	0.46	Primary	LR MC Continuous	1.35	1.06	1.71	0.014
		Primary	LR MC Dominant	1.97	1.28	3.04	0.002
		Combined	LR MC Dominant	1.48	1.14	1.92	0.003
		Primary	LL Case effect, dominant	0.53	0.30	0.93	0.026
		Combined	LL TDT	0.80	0.65	0.99	0.039
		Combined	LL Case effect, dominant	0.60	0.42	0.86	0.005
*TCblR* rs2336573 G220R	0.04	Combined	LR CC Dominant	.^***^			0.013

^*^For each SNP, the minor allele was evaluated as the risk allele.

^**^ Primary: 320 triads, 341 controls. Combined: 570 triads, 999 controls.

^***^ (.) = Failure to converge. P_exact_ is shown.

## Conclusions

In summary, this study involves the largest evaluation of common genetic variation for NTD risk yet reported: 1441 SNPs in 82 candidate genes. While no SNP associations remained significant after correction for multiple tests, there is a strong possibility that the study design and/or stringency of correction has resulted in obscuring true associations. At least one established risk factor, *MTHFD1* R653Q, was corrected away, suggesting our approach was extremely conservative. Therefore, variation in the top genes identified in this study should be examined in independent populations for NTD risk, especially since many of these genes (*MFTC, CDKN2A, ADA, CUBN*, *DNMT3A*, and *T (Brachyury)*) represent new avenues of investigation.

## Methods

### Study population

The recruitment of the Irish NTD families (cases and parents) and controls has been described [23,34,51,52]. Briefly, the cohort includes 586 families with an NTD case; 442 of these families are full family triads (DNA from case, mother and father). For this study, 570 of the NTD families had sufficient DNA and were divided into two sets, one for primary analysis and one for secondary (combined) analysis. The primary and secondary sample sets were matched as closely as possible for the following parameters: the number of complete NTD triads, the proportion of NTD cases with spina bifida vs. other NTDs, and NTD case gender (Table 6).

**Table 6:** Characteristics of the Irish NTD Sets Used in this Study

	**No. Families (%)**	**No. Cases (%)**	**% Complete Triads**	**% Spina Bifida**	**% Female Cases**
**Primary set**	320 (56.1%)	301 (56.8%)	75.2%	94.9%	51.8%
**Secondary set**	250 (43.9%)	229 (43.2%)	73.6%	95.7%	55.3%

The control population (n = 999) is a random sample drawn from 56,049 blood samples donated by women at their first prenatal visit to the three major maternity hospitals in Dublin (1986–1990). A subset of controls (n = 341) was randomly selected for the primary screen, and the remaining controls were used in the secondary set.

Written, informed consent was obtained from study subjects, their parents or their guardians. Archived control samples were anonymized prior to analysis. The study was approved by the Ethics Research Committee of the Health Research Board (Dublin, Ireland) and the Institutional Review Board at the National Human Genome Research Institute (Bethesda, MD, USA). Extraction of genomic DNA from blood samples and buccal swabs was performed using the QIAamp DNA Blood Mini Kit (Qiagen, Valencia, CA, USA).

### SNP selection

Genes were chosen for study because they are involved in folate and vitamin B12 metabolism or other metabolic and signaling pathways implicated in the etiology of NTDs (Figure 1). Although previously published, *MTHFR*[52-54], *MTHFD1*[23,25], *MTHFD1L*[27], *TCblR*[34], and *TP53*[51] were reanalyzed in this study in order to compare the differing research strategy, which includes new genetic models of risk assessment. For each gene chosen, we evaluated the transcribed region of the gene and 10 kilobases (kb) upstream and downstream of the gene in an effort to include polymorphisms with potential proximal effects, such as promoter variants. In order to capture the common variation in each gene, SNPs genotyped by HapMap (Data Release 21, Phase II, Anon, 2003 [55]) were considered. A set of tagSNPs was identified using an algorithm based on optimizing for tagSNPs with maximal minor allele frequencies (MAFs) and an r^2^ threshold of 0.8, while maximizing the MAF of the selected tagSNP. In addition to this set of tagSNPs, validated variants from dbSNP [26] were also selected for physical coverage so that spacing between SNPs would be less than 20 kb within D’ haplotype blocks and less than 5 kb between haplotype blocks. A total of 1517 SNPs were selected.

### Genotyping

The selected 1517 SNPs were genotyped on the primary sample sets (320 NTD case families and 341 controls). Genotypes were generated by the Johns Hopkins University SNP Center (Baltimore, MD) using the Illumina GoldenGate assay (Illumina, San Diego, CA). Of the 1517 SNPs attempted, 1320 SNPs (87.2%) remained after filtering out low quality data (re-attempted on another platform, see below). The overall call rate for these 1320 SNPs was 98%. All but 150 of these SNPs had a call rate of >95%; the rest had an average call rate of 87.2% (±7.9%) and were re-genotyped on another platform (see below). Both the overall duplicate concordance rates and the Mendelian consistency rates were 99.99% for the 1320 accepted SNPs.

Based on analyses of the primary sample sets, 93 SNPs were genotyped in the secondary sample set (250 NTD case families and 658 controls). Genotypes were generated by KASPar chemistry at Kbiosciences (Herts, UK). Three SNPs failed on this platform: rs127317149, rs7367859 and rs7096079. For the 90 successfully genotyped SNPs, the overall duplicate concordance rate was 99.81% and the overall Mendelian consistency rate was 99.94%.

SNPs that could not be assayed (n = 197) or returned low call rates (<95%, n = 150) via Illumina GoldenGate chemistry were re-genotyped in the entire sample set (570 NTD families and 999 controls) by detection of allele-specific primer extension using matrix-assisted laser desorption/ionization – time of flight (MALDI-TOF) mass spectrometry (Sequenom, San Diego, CA, USA). Genotyping data from SNPs with call rates above 95% were added to the final analyses (121/197 SNPs that failed and 106/150 SNPs that yielded low call rates). The overall duplicate concordance rate was 99.10% and the overall Mendelian consistency rate was 99.32% for these 227 SNPs.

To summarize, our final data set consisted of 1441 SNPs; 1320 high quality SNPs from the Illumina platform (93 of these SNPS were also typed in the secondary samples using the KASPar platform, and 106 of these SNPs were re-typed in the entire sample set using the Sequenom platform) plus 121 SNPs from the Sequenom platform.

### Linkage disequilibrium

D’ and r^2^ measures of LD for SNPs of interest were estimated based on control genotypes using Haploview [56]. Haplotype blocks were based on D’ values using the Solid Spine of LD option in Haploview.

### Statistical methods

The design for this study involved two stages of genotyping. Rather than use the secondary samples for a replication study, joint analysis of the combined dataset was performed since it generally provides greater power to detect a genetic effect [57]. In the initial analysis, all SNPs successfully typed with the Illumina GoldenGate assay (n = 1320, including the 150 high quality SNPs with call rates of <95%) were analyzed in the primary sample set by four tests of association. Two tests were performed to evaluate case effects: 1) Logistic regression, a 1-degree of freedom (DOF) test of association between the affected status and number of risk alleles; and 2) the Spielman transmission disequilibrium test (TDT, [58]). Similarly, two tests were performed to evaluate maternal effects: 1) Logistic regression, a 1-DOF test of association between the maternal status and number of risk alleles; and 2) log-linear modeling with 2-DOF to test for effect of the maternal genotype.

SNPs were selected to be genotyped on all samples (570 NTD case families and 999 controls) if they met the following criteria: 1) SNPs of interest reaching a significance level in the primary analysis (n = 93, Table 2); or 2) failed SNPs (n = 121) and SNPs with low call rates (<95%, n = 106). Final analyses were performed on the entire dataset and consisted of twelve association tests. NTD case–control and NTD mother-control comparisons were performed using continuous, recessive and dominant coded models of logistic regression to generate odds ratios (ORs) and 95% confidence intervals (CI). Six family-based tests of association were also applied using log-linear models. The NTD triads (case, mother and father) were analyzed for case effects using recessive, dominant and linear (TDT) coding while direct maternal effects were analyzed using recessive, dominant and 2-DOF models.

To correct for multiple comparison, the final analysis used the complete information on the 1441 SNPs. Correction was performed by multivariate permutation (N = 9,999 random permutations) for three of the tests used in the initial analysis: case–control logistic regression, mother-control logistic regression and the TDT. This method accounts for any linkage disequilibrium between SNPs. Multivariate permuting of triads for the TDT was performed by treating the test as a one-sample test and permuting the risk allele [59]. Independent permutations were performed for the cases and controls or mothers and controls to adjust for multiple comparisons in the tests of logistic regression. The results were combined by Bonferroni adjustment to account for all comparisons (including all SNPs because of our multivariate permutation approach) while controlling the probability of any false positives at 5%. Since a SNP could only be found significant based on this combined analysis, our method provides type I error control regardless of the SNP selection process or number of SNPs selected for genotyping on all samples.

## Abbreviations

DOF, Degree(s) of freedom; LD, Linkage disequilibrium; NTDs, Neural tube defects; RAF, Risk allele frequency; SNP, Single nucleotide polymorphism; TDT, Transmission disequilibrium test.

## Competing interests

The authors declare that they have no competing interests.

## Authors’ contributions

FP, AMM, JLM, PNK, BS, JMS and LCB formulated the study design and performed candidate gene selection. FP additionally performed SNP selection and drafted the manuscript. AMM additionally managed the DNA samples and their selection. AP-M, VBO’L and CS participated in candidate gene selection. JMS and KG-S prepared DNA. AM and JEV extracted DNA and performed genotyping. KMK, AS, JC-H and NS performed genotyping. PC developed the SNP selection algorithm. MC provided database management. JFT contributed to study design and performed the statistical analyses. All authors read, edited and approved the final manuscript.

## Pre-publication history

The pre-publication history for this paper can be accessed here:

http://www.biomedcentral.com/1471-2350/13/62/prepub
